# Trained immunity: New insights into pathogenesis and therapeutic targets in diabetes and diabetic complications

**DOI:** 10.1016/j.gendis.2025.101940

**Published:** 2025-11-19

**Authors:** Qiming Gong, Yuqing Huang, Fahui Liu, Tingting Zhou, Wei Huang, Yong Xu

**Affiliations:** a Department of Endocrinology and Metabolism, The Affiliated Hospital of Southwest Medical University, Luzhou, Sichuan 646000, China; b Metabolism, Metabolic Vascular Diseases Key Laboratory of Sichuan Province, Luzhou, Sichuan 646000, China; c Sichuan Clinical Research Center for Nephropathy, Luzhou, Sichuan 646000, China; d Sichuan-Chongqing Joint Key Laboratory of Metabolic Vascular Diseases, Luzhou, Sichuan 646000, China; e Department of Nephrology, Affiliated Hospital of Youjiang Medical University for Nationalities, Key Laboratory of Medical Research Basic Guarantee for Immune-Related Diseases Research of Guangxi, Baise, Guangxi 533000, China; f Xiamen Cell Therapy Research Center, The First Affiliated Hospital of Xiamen University, School of Medicine, Xiamen University, Xiamen, Fujian 361005, China

**Keywords:** Diabetes mellitus, Epigenetics, Immunometabolism, Innate immune system, Trained immunity

## Abstract

Diabetes mellitus, a chronic metabolic condition, is marked by ongoing hyperglycemia and poses an increasing global health issue. Beyond its recognized contribution to the development of cardiovascular diseases and kidney problems, diabetes can profoundly impact immune system functions. Recent developments in immunology have revealed trained immunity as a mechanism through which innate immune cells experience enduring functional modifications following their first encounter with specific stimuli. This review compiles the latest evidence concerning the role of trained immunity in the development of diabetes and its complications. Moreover, it discusses emerging therapeutic opportunities that may arise from modulating trained immunity pathways. This review emphasizes the complex relationship between metabolic dysregulation and innate immune memory by synthesizing results from various studies. It proposes that focusing on trained immunity may provide innovative approaches for managing diabetes and its related complications.

## Introduction

Diabetes mellitus encompasses metabolic conditions distinguished by ongoing hyperglycemia, which arise from issues related to insulin secretion, its effectiveness, or a combination of these factors. Around 589 million adults are anticipated to be living with diabetes in 2024, with this figure likely to rise to 853 million by 2050.^1^ This persistent elevation in blood glucose levels poses a substantial global health burden, contributing not only to increased morbidity but also to elevated mortality rates. Extensive evidence has demonstrated the strong association between uncontrolled diabetes and the development of severe complications. Inadequate management of blood sugar levels greatly heightens the likelihood of developing cardiovascular conditions, which remain the primary causes of mortality among people with diabetes.^2^^,^^3^ Furthermore, diabetic kidney disease is critical in end-stage renal disease, whereas diabetic neuropathy frequently results in complications in the lower extremities, including foot ulcers and, in more severe instances, amputations of limbs.^4^ Given these significant consequences, addressing the urgent requirement for effective strategies to prevent and manage complications associated with diabetes is essential.^5^^,^^6^ Beyond the direct health impact, the complications associated with diabetes place a considerable burden on public healthcare systems, along with substantially diminishing life quality for affected individuals.^7^

**Figure 1:**
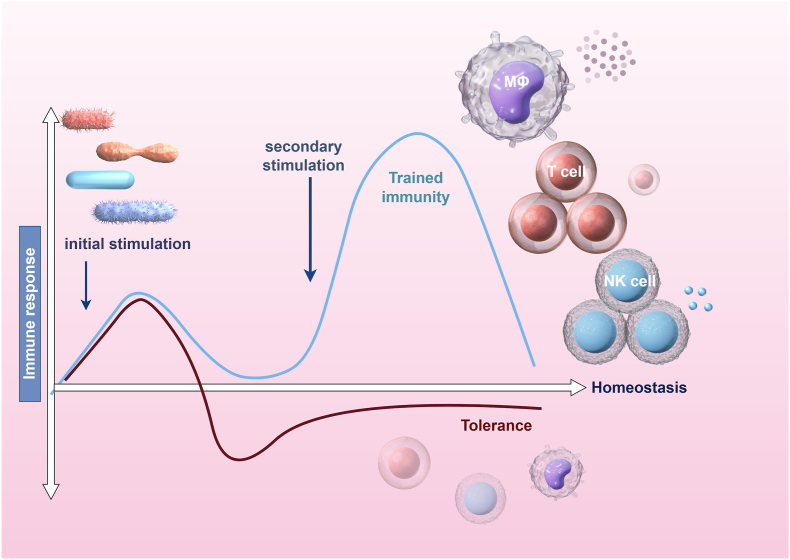
Mechanisms of inflammatory activation and trained immune responses. Microbial or endogenous stimuli initiate a primary response by activating innate immune cells. Depending on the strength and duration of stimulation, this activation may trigger regulatory mechanisms that induce tolerance, preventing excessive inflammation upon re-exposure. Upon subsequent stimulation, the cells do not elicit another pro-inflammatory response, thereby preserving tissue integrity by limiting inflammatory damage. This image was created using Figdraw.com.

Recent developments in immunology have challenged the conventional perspective that regards the innate immune system as a non-adaptive element of host defense. Innate immunity refers to immediate, non-specific defense mechanisms that do not confer long-term memory, whereas adaptive immunity involves antigen-specific responses by T and B cells leading to immunological memory. Trained immunity (TI) represents a recently recognized intermediate phenomenon in which innate immune cells undergo long-lasting reprogramming after an initial challenge, resulting in an enhanced response upon re-stimulation. TI is distinct from general acute inflammation in that it endows innate cells with a memory-like state persisting weeks to months, unlike classical transient inflammation, which resolves once the trigger is removed. TI pertains to the enduring functional reprogramming of innate immune cells after encountering an initial stimulus.^8^ Notably, Experimental models commonly used to study TI include *in vitro* stimulation of human peripheral blood mononuclear cells or monocytes with microbial ligands, such as β-glucan^9^ or Bacillus Calmette–Guérin (BCG),^10^ as well as *in vivo* models utilizing BCG vaccination^11^ or low-dose lipopolysaccharide preconditioning in mice.^12^ Trained innate immune cells exhibit hallmark changes, such as enhanced glycolysis, accumulation of metabolites like succinate, and persistent chromatin modifications (*e.g.*, *H3K4me3* and *H3K27a*c at inflammatory gene loci) and increased production of pro-inflammatory mediators (IL-1β, IL-6, TNF-α) upon re-stimulation that together prime these cells for robust secondary responses. This concept has gained particular relevance in explaining chronic inflammation associated with diabetes. In contrast to adaptive immunity, which targets specific antigens and offers prolonged defense, TI engages innate immune cells like monocytes and macrophages. When first exposed to a stimulus, these cells undergo functional reprogramming, leading to enhanced effector responses upon re-stimulation, a process defined as TI. Both TI and immune tolerance represent distinct phenotypes of innate immune memory that are primarily influenced by metabolic and epigenetic reprogramming. The results of these processes are contingent upon the characteristics of the initial stimulus (Fig. 1).^13^ TI results in a sustained baseline change in innate cells (marked by enduring epigenetic and metabolic reprogramming), whereas classical M1/M2 macrophage polarization is an acute, reversible response to a particular cytokine milieu. TI's enhanced inflammatory capacity is not simply the same as an M1 phenotype; rather, TI can skew macrophage polarization in a long-term manner beyond the immediate stimulus. Recent studies have clarified the mechanisms underlying TI, including alterations in cellular metabolism, epigenetic modifications, and regulation by cytokines and signaling molecules.^8^ Hyperglycemia has been shown to push monocytes into a pro-inflammatory condition, consequently heightening the likelihood of cardiovascular issues in individuals with diabetes.^14^ TI may be influenced by gut microbiota, suggesting that changes in microbial composition could affect immune function and metabolic health.^15^ Investigating TI provides promising therapeutic opportunities. By targeting its core mechanisms, it may be possible to improve blood glucose control while addressing inflammation that contributes to diabetic complications. For instance, dietary interventions (Mediterranean diet) have been reported to positively impact immune and metabolic functions.^16^ Additionally, several pharmacological agents, including statins and anti-inflammatory drugs, are currently under investigation for their capacity to modulate TI and alter the course of diabetes complications.^17^ Recent research also indicates that TI could play a role in safeguarding against insulin resistance and other metabolic issues linked to diabetes.^18^

In summary, the concept of TI is reshaping the understanding of the immune system's role in diabetes and its associated complications. By integrating insights from immunology, metabolism, and clinical research, we can better understand how immune dysregulation and metabolic disturbances are interconnected in diabetes. Herein, this review seeks to encapsulate the latest discoveries regarding TI, examine its effects on the advancement and evolution of diabetes, and pinpoint potential therapeutic targets based on this emerging knowledge.

## Mechanisms of trained immunity

**Figure 2:**
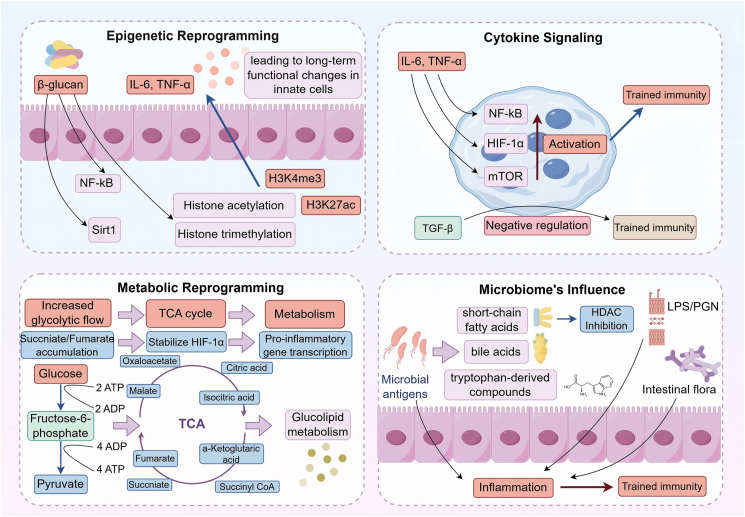
Mechanisms of trained immunity. Exogenous stimuli (*e.g.*, β-glucan, LPS) engage pattern-recognition receptors and rewire cellular metabolism. The resulting metabolites act as intermediates—not stimuli—that feed back to chromatin (*e.g*., succinate stabilizes *HIF-1α*; acetyl-CoA supports *H3K27ac*), driving epigenetic remodeling. These durable metabolic–epigenetic changes imprint a trained state, yielding heightened cytokine production and antimicrobial responses upon restimulation. The solid arrows denote receptor-proximal signaling, and dashed arrows denote metabolite-to-chromatin effects. The figure was created with Figdraw.com.

TI signifies the enduring functional reprogramming of innate immune cells, including monocytes, macrophages, and natural killer (NK) cells, through epigenetic and metabolic modifications (Fig. 2). Upon encountering specific stimuli, such as pathogens or vaccines, these cells undergo changes that enable them to mount an enhanced response to subsequent challenges.

### Epigenetic reprogramming

Epigenetic modifications are crucial in the progression of TI. Changes such as histone acetylation and DNA methylation can influence gene expression by enhancing chromatin accessibility. TI encompasses particular alterations, notably enhanced histone acetylation (*e.g.*, *H3K27ac*) and methylation (*e.g.*, *H3K4me3*), which facilitate the expression of inflammatory cytokines.^19^ Research indicates that β-glucan, found in the fungal cell walls, triggers these alterations by stimulating transcription and boosting inflammatory signaling.^9^ β-glucan additionally stimulates *SIRT1*, an enzyme that modifies histones and changes acetylation patterns, leading to a heightened level of pro-inflammatory cytokines (*IL-6* and *TNF-α*).^20^ Moreover, β-glucan enhances the activity of nuclear factor-kappa B (*NF-κB*), a transcription factor that stimulates the inflammatory gene expression and recruits enzymes that modify chromatin structure.^21^ New findings suggest that these epigenetic shifts can persist long after the initial exposure, establishing a type of memory within innate immune cells.^22^^,^^23^ This highlights the potential of targeting epigenetic mechanisms as a viable approach to improve vaccine efficacy and address infectious or inflammatory conditions. Further research into these mechanisms may support the development of novel immune-based therapies.

### Metabolic reprogramming

Metabolic reprogramming is a fundamental component of TI, reflecting a shift in the metabolic pathways utilized by innate immune cells following activation. A notable change that has been identified is the transition from oxidative phosphorylation to aerobic glycolysis, often known as the Warburg effect. This change in metabolism results not just from immune activation but also functions as an essential adaptation, providing energy and the necessary biosynthetic precursors for generating pro-inflammatory mediators. For example, activated macrophages demonstrate increased glycolytic activity, resulting in a rise in ATP production. This process also causes the buildup of metabolic intermediates that act as signaling molecules, thereby facilitating inflammation-related gene expression.^24^ The coordination between glycolysis and other metabolic pathways (tricarboxylic acid cycle) is pivotal in shaping immune responses. Specific tricarboxylic acid cycle-derived metabolites, such as succinate and itaconate, can act as epigenetic regulators by influencing histone modifications and transcription factor activity, thereby promoting a pro-inflammatory phenotype in trained innate immune cells.^25^^,^^26^ Additionally, there is a growing fascination with the significance of fatty acid metabolism in TI, with evidence suggesting that fatty acid oxidation leads to inflammatory response enhancement and may support the long-term functional changes associated with TI.^27^ Taken together, these results emphasize that the metabolic condition of innate immune cells mirrors their activation level and influences their ability to maintain immune responsiveness when re-exposed.

### Cytokine signaling

Cytokine signaling is crucial for both the establishment and ongoing maintenance of TI, allowing innate immune cells to respond robustly after their initial activation. Inflammatory cytokines (*IL-1β* and *TNF-α*) enhance the inflammatory potential of these immune cells by triggering signaling pathways that activate transcription factors like *NF-κB* and hypoxia-inducible factor 1-alpha (HIF-1α). These transcription factors are essential for enhancing inflammation-related gene expression.^28^ Additionally, cytokine-mediated activation of the mechanistic target of rapamycin (*mTOR*) pathway meets the metabolic needs of trained immune cells by facilitating both glycolysis and oxidative phosphorylation. This process supplies the energy and substrates required for prolonged immune function.^29^, ^30^, ^31^ Conversely, transforming growth factor-β (*TGF-β*) functions as an inhibitor of NK cell activation and activity by inhibiting the *mTOR* signaling pathway. This inhibition impairs NK cell metabolism and promotes a functionally suppressed state, which may facilitate immune evasion in the tumor microenvironment.^32^ *TGF-β* attenuates the immediate activity of NK cells and reduces their capacity for long-term immune responses through metabolic reprogramming.^33^, ^34^, ^35^, ^36^ While NK cells fall under the category of innate lymphoid cells, they can demonstrate memory-like characteristics when certain conditions are met. For example, brief exposure to a mix of interleukin-12 (*IL-12*), *IL-15*, and *IL-18* may trigger improved functional responses upon subsequent stimulation.^37^ The interaction between cytokine signaling and epigenetic remodeling is now recognized as a fundamental mechanism in sustaining TI, as these modifications can lead to persistent changes in gene expression that amplify inflammatory responses over time.^38^

## The microbiome's influence

The gut microbiome is significant in shaping TI through multiple interconnected mechanisms, including the production of microbial metabolites, direct interactions with immune cells, and epigenetic regulation. One of the extensively researched mechanisms pertains to short-chain fatty acids like butyrate, acetate, and propionate, which arise from the microbial fermentation of dietary fibers. These short-chain fatty acids inhibit histone deacetylases, which results in chromatin remodeling and modifications in gene expression within innate immune cells, especially macrophages and monocytes. This process enhances their ability to respond to subsequent stimuli.^39^^,^^40^ In addition to short-chain fatty acids, other metabolites (*e.g.*, secondary bile acids) and tryptophan derivatives (*e.g.*, indole compounds) have also been associated with TI. They may affect immune receptor signaling and modulate cytokine production, including *IL-1β* and *IL-22*, thereby further refining innate immune responses.^41^ In addition to metabolic byproducts, microbial components, such as lipopolysaccharides (LPS) and peptidoglycans, engage pattern recognition receptors, including Toll-like receptors (TLRs), on innate immune cells. These interactions trigger signaling cascades that promote epigenetic reprogramming and metabolic shifts, notably enhancing glycolysis and reactive oxygen species (ROS) production, thereby increasing antimicrobial capacity.^11^ Notably, certain commensal and environmental bacteria, such as Bacillus subtilis and specific *Escherichia coli* strains, have been shown to act as potent inducers of TI, priming the innate immune system for more effective responses to subsequent, unrelated pathogens.^42^^,^^43^ Conversely, microbial imbalance, or dysbiosis, has been associated with metabolic disorders, including diabetes, suggesting that disruptions in microbiome composition may impair TI and contribute to disease pathogenesis.^44^^,^^45^

## Factors that trigger diabetes

### Metabolic dysregulation and advanced glycation end products (AGEs)

Chronic nutrient excess and obesity are early, canonical drivers of diabetes pathogenesis. Even before overt hyperglycemia, adipose tissue becomes infiltrated by pro-inflammatory macrophages and other innate immune cells, creating a state of low-grade “metaflammation” that impairs insulin signaling in the liver, muscle, and fat. Importantly, this nutrient-excess milieu can prime innate immune cells toward a trained phenotype, predisposing to exaggerated responses upon secondary challenges and thereby accelerating metabolic dysfunction.^46^ Notably, saturated free fatty acids such as palmitic acid (often elevated in obesity) activate Toll-like receptors (particularly *TLR4*) on innate immune cells, provoking inflammatory responses and insulin resistance. This *TLR4* activation also promotes the induction of TI, characterized by increased cytokine production and enhanced phagocytic activity. Additionally, chronic high blood sugar levels, a defining characteristic of diabetes mellitus, have been demonstrated to trigger TI in innate immune cells.^47^ Increased glucose levels enhance pro-inflammatory gene expression and promote atherogenic phenotypes in macrophages through increased glycolytic activity. Notably, macrophages isolated from the bone marrow of diabetic mice retained these inflammatory traits even under normal glucose conditions, suggesting that chronic hyperglycemia may lead to a form of innate immune memory consistent with TI.^14^ In individuals with diabetes, elevated blood glucose is correlated with higher endothelial activation marker levels, including E-selectin, soluble cell adhesion molecule-1 (sCAM-1), and vascular cell adhesion molecule-1 (VCAM-1), which may promote leukocyte adhesion to vascular endothelium.^48^ Simultaneously, elevated blood sugar levels promote the development of AGEs, which stimulate ROS production and a subsequent rise in the expression of adhesion molecules.^49^ AGEs are formed through the non-enzymatic processes of glycation and oxidation, affecting proteins and lipids, and they often build up in the tissues and serum of people suffering from diabetes. These compounds interact with receptors like the receptor for advanced glycation end products (RAGE) on innate immune cells, activating intracellular pathways involving *NF-κB* and mitogen-activated protein kinase (*MAPK*) signaling cascades.^50^, ^51^, ^52^ This activation results in epigenetic reprogramming and metabolic changes that favor glycolysis, both of which contribute to the development of TI. Specific AGEs, such as Nε-(carboxymethyl)lysine, have been reported to increase cytokine (*IL-1β* and *TNF-α*) production in macrophages following a secondary stimulation.^53^ Diabetes is also commonly associated with lipid abnormalities, including elevated free fatty acids and altered lipoprotein profiles. Saturated free fatty acids such as palmitic acid activate TLRs (particularly TLR4) on innate immune cells, leading to inflammatory responses and insulin resistance. Saturated free fatty acids such as palmitic acid have been reported to activate *TLR4* on innate immune cells, leading to inflammatory responses and insulin resistance. However, recent evidence suggests that palmitate may not directly bind to TLR4; rather, TLR4 signaling is required for palmitate-induced inflammation through indirect mechanisms of metabolic reprogramming.^54^ Despite this nuance, *TLR4* engagement by metabolic stimuli can promote the induction of TI, characterized by increased cytokine production and enhanced phagocytic activity.^55^^,^^56^ In addition, oxidized low-density lipoprotein that builds up in atherosclerotic plaques activates macrophages through scavenger receptors and encourages a pro-inflammatory variant of TI. This mechanism may accelerate atherosclerosis in individuals with diabetes.^57^^,^^58^

### Microbial factors

The gut microbiome significantly influences various metabolic and inflammatory conditions, including obesity, non-alcoholic fatty liver disease, insulin resistance, and chronic inflammation, all intricately linked to the onset of type 2 diabetes (T2D).^59^^,^^60^ In cases of T2D, dysbiosis of the gut is frequently noted, marked by changes in microbial diversity and heightened intestinal permeability.^61^, ^62^, ^63^ This imbalance promotes the movement of microbial elements, including LPS and peptidoglycans, into the systemic circulation. Among these microbial products, LPS is a potent agonist of *TLR4* and a known inducer of TI. Chronic exposure to low levels of LPS, as seen in dysbiosis, can prime innate immune cells and contribute to sustained inflammation, thereby aggravating diabetes-related complications, such as nephropathy and cardiovascular disease.^64^^,^^65^ Employing linear discriminant analysis effect size (LEfSe), researchers discovered notable variations in 43 bacterial taxa when comparing Chinese T2D patients to healthy controls: *Acidaminococcales*, *Bacteroides plebeius*, and *Phascolarctobacterium* sp. CAG207 has been suggested as a possible biomarker for T2D.^66^ *Lactobacillus* species have been associated with improved fasting glucose levels and lower glycosylated hemoglobin (HbA1c), whereas *Clostridium* species show negative correlations with glycemic markers and plasma triglycerides. Individuals newly diagnosed with T2D exhibited elevated levels of *Lactobacillus*, accompanied by reduced levels of *Clostridium coccoides* and *Clostridium leptum*.^67^ Also, those with diabetes tend to be more prone to infections, with chronic or recurrent infections serving as potent inducers of TI. Fungal infections, particularly those caused by *Candida albicans*, have been shown to induce long-lasting functional changes in monocytes, enhancing cytokine production and antifungal responses. The N-linked mannans of *C. albicans* activate TI by engaging the dectin-2 receptor.^68^

## Trained immunity in the pathogenesis of diabetes

### Inflammation and insulin resistance

Chronic low-level inflammation is increasingly acknowledged as a significant contributor to insulin resistance and the progression of T2D.^69^ This persistent state of inflammation typically begins in adipose tissue and the gastrointestinal tract, hindering insulin signaling in crucial metabolic organs like the liver, skeletal muscle, and fat tissue. Consequently, the processes of glucose uptake and utilization become impaired, leading to widespread metabolic dysfunction. In individuals with obesity, innate immune cells exhibit features of enhanced TI, responding more aggressively to stimuli such as saturated fatty acids and LPS, which can lead to increased *TNF-α*, *IL-1β*, and *IL-6*.^57^^,^^70^ The insulin signaling is disrupted by these cytokines, which activate inflammatory pathways including *NF-κB* and c-Jun N-terminal kinase (*JNK*). As a result, serine phosphorylation of insulin receptor substrate-1 (IRS-1) occurs. This modification disrupts its interaction with the insulin receptor, thereby contributing to insulin resistance.^71^, ^72^, ^73^ Metabolic stress associated with obesity further exacerbates inflammation by increasing endoplasmic reticulum stress and oxidative stress.^74^^,^^75^ Endoplasmic reticulum stress triggers inflammatory signaling pathways, while oxidative stress contributes to ROS accumulation. This, subsequently, activates NOD-like receptor, pyrin domain-containing 3 (*NLRP3*) inflammasomes, leading to caspase-1 activation and eventual maturation and release of *IL-1β*.^76^ Moreover, monocytes from individuals with obesity display an exaggerated inflammatory response upon stimulation, consistent with the presence of insulin resistance.^77^

### Impaired β-cell function

Type 1 diabetes (T1D), marked by the immune system attacking pancreatic β-cells, leads to complete loss of insulin production. While this destruction is primarily driven by autoreactive T cells of the adaptive immune system, the innate immune system and TI also play important roles.^78^^,^^79^ β-cells express TLRs, and activation of these receptors by ligands such as LPS or viral RNA can directly impair β-cell function. Prior exposure to certain TLR ligands can prime innate immune cells, leading to epigenetic and metabolic changes that cause an exaggerated cytokine release upon subsequent stimulation. Such heightened responses can exacerbate β-cell stress and dysfunction.^80^^,^^81^ Chronic high blood sugar levels and increased free fatty acids can trigger *NLRP3* inflammasome activation in both immune and β-cells. Following this activation, the release of *IL-1β* can hinder the functionality of β-cells and encourage their death. The presence of TI may exacerbate this activation, as previous metabolic stress prepares innate immune cells to respond more vigorously to subsequent stimuli, resulting in heightened *IL-1β* production and further deterioration of β-cell function.^82^ Continuous exposure to elevated glucose and free fatty acids leads to endoplasmic reticulum stress in β-cells. While the unfolded protein response initially attempts to restore normal function, prolonged endoplasmic reticulum stress can ultimately result in β-cell dysfunction and apoptosis.^83^^,^^84^ It is conceivable that TI could amplify these harmful effects—for instance, metabolically “trained” innate immune cells may release greater amounts of inflammatory cytokines under diabetic stress, potentially accelerating β-cell failure—although direct evidence for this mechanism has yet to be established.

Recent studies suggest TI in macrophages could participate in systemic insulin resistance and pancreatic β-cell dysfunction. Macrophages exhibit a dual role in inflammation by promoting or resolving immune responses depending on their polarization state. M1 macrophages, which are classically activated, generate pro-inflammatory cytokines and are linked to tissue damage. In contrast, M2 macrophages, known for their alternative activation, facilitate tissue repair and help resolve inflammation. TI may influence macrophage polarization, favoring a shift toward the M1 phenotype. This imbalance fosters a pro-inflammatory environment within the pancreas, exacerbating β-cell damage. The resulting inflammatory state further impairs insulin signaling and β-cell function, creating a self-perpetuating cycle that accelerates diabetes progression.^85^, ^86^, ^87^ Ieronymaki and their team showed that extended exposure of macrophages to insulin, both in laboratory settings and in living mice with glucose intolerance induced by diet, results in insulin resistance, which is indicated by decreased phosphorylation of V-akt murine thymoma viral oncogene homolog 2 (*Akt2*). Notably, macrophages deficient in *Akt2* or the *IGF1* receptor exhibited a similar insulin-resistant phenotype.^88^ Furthermore, Kaur and her team emphasized the intricate regulatory nature of circular RNAs (circRNAs) in the immune-driven destruction of β-cells associated with *T1D*, highlighting their possible involvement in disease pathogenesis.^89^ In summary, metabolic stressors (obesity, nutrient excess, hyperglycemia, and dyslipidemia) can instigate TI in adipose tissue and circulating innate cells early in disease, amplifying cytokine output upon subsequent challenges. This trained state contributes to insulin resistance in classical metabolic organs and imposes inflammatory stress on pancreatic β-cells, creating a feed-forward loop that accelerates diabetes progression.

## Diabetic complications

### Atherosclerosis

TI is gaining recognition as a significant contributor to the development of complications related to diabetes, including atherosclerosis. In the atherosclerotic environment, macrophages from diabetic patients display an enhanced TI phenotype, characterized by increased lipid uptake, elevated foam cell formation, and heightened secretion of pro-inflammatory mediators. These changes lead to instability in plaque formation and increase the likelihood of cardiovascular incidents.^90^ In particular, TI triggered by oxidized low-density lipoprotein has been linked to the advancement of atherosclerosis related to diabetes.^91^ Macrophages that undergo training by oxidized low-density lipoprotein exhibit exaggerated inflammatory responses, producing elevated levels of *IL-1β*, *IL-6*, and *TNF-α*. This enhanced cytokine production not only destabilizes atherosclerotic plaques but also increases matrix metalloproteinase (MMP) activity and reduces collagen content, thereby raising the likelihood of plaque rupture and thrombotic complications.^92^, ^93^, ^94^ Recent research has highlighted the significance of TI in enhancing vascular inflammation through the activation of the *NLRP3* inflammasome within macrophages. This activation leads to endothelial dysfunction and reduced vascular responsiveness, exacerbating atherosclerosis, especially in individuals with diabetes.^95^^,^^96^ Yalcinkaya and his team have shown that BRCC3 aids in the deubiquitylation of *NLRP3*, thereby facilitating inflammasome activation and the advancement of atherosclerosis, particularly in cases of Tet2 clonal hematopoiesis.^97^

### Diabetic kidney disease

In addition to atherosclerosis, TI also contributes to the development of diabetic kidney disease. Renal macrophages that have undergone TI infiltrate the kidney and secrete elevated amounts of pro-inflammatory cytokines and chemokines, contributing to glomerular injury, proteinuria, and progressive renal fibrosis.^98^ Hyperglycemia-induced TI enhances the expression of profibrotic mediators like *TGF-β* and connective tissue growth factor (CTGF), promoting extracellular matrix accumulation and fibrotic remodeling, hallmarks of diabetic kidney disease.^99^ Additionally, TI intensifies *NLRP3* inflammasome activation within renal cells, increasing secretion of *IL-1β* and directly hindering podocyte function, which worsens proteinuria.^100^ Research has demonstrated that *IL-17A* exacerbates podocyte damage by promoting *IL-1β* production through the ROS–*NLRP3* inflammasome-caspase-1 axis.^101^ Recent studies have also underscored the importance of histone modifications, including *H3K4me3* and *H3K27ac*, in preserving the epigenetic programming of trained renal macrophages, thus ensuring their pro-inflammatory characteristics are sustained over time.^102^^,^^103^

### Wound healing

While TI can enhance host defense against infections, it may also negatively impact wound healing in individuals with diabetes. The exaggerated inflammatory response driven by trained macrophages can disrupt the finely tuned balance of growth factors and matrix remodeling enzymes necessary for proper tissue repair. Additionally, trained macrophages may exhibit impaired efferocytosis or the clearance of apoptotic cells, further delaying wound resolution.^104^ Shrestha et al showed that TI contributes to the priming of neutrophil extracellular traps induced by diabetes, which negatively affects wound healing in diabetic environments.^105^ In conditions of hyperglycemia, neutrophils experience considerable metabolic reprogramming, marked by an increase in glycolytic activity through the pentose phosphate pathway and a boost in fatty acid oxidation. This leads to acetyl-coenzyme A buildup, which, through ATP-citrate lyase, facilitates histone acetylation at particular sites, such as *H3K27ac* and *H4K8ac*, thereby contributing to prolonged pro-inflammatory responses that obstruct tissue repair.^106^^,^^107^

### Diabetic retinopathy

The regulatory function of post-translational modifications in histone proteins is vital for diabetic retinopathy development. Under persistent hyperglycemia or poor glycemic control, levels of histone marks such as *H3K4me1* and *H3K4me2* are reduced in retinal tissue. This reduction persists even after blood glucose normalization and is associated with sustained suppression of *Sod2*, a gene encoding mitochondrial superoxide dismutase.^108^ In diabetic rodent models, post-translational modifications affecting the transcription factor nuclear factor erythroid 2-related factor 2 (*Nrf2*) have also been shown to impair antioxidant defense mechanisms during the progression of retinopathy.^109^ Moreover, the pro-oxidative molecule thioredoxin-interacting protein (*TXNIP*) is up-regulated in retinal endothelial cells under high-glucose stimulation. This up-regulation promotes inflammation through epigenetic alterations, including increased histone H3K9 acetylation (*H3K9ac*) and decreased H3K9 trimethylation (*H3K9me3*), thereby contributing to retinal dysfunction.^110^^,^^111^ Notably, most retinal studies described here reflect persistent “metabolic memory” of resident cells rather than classical TI in innate immune cells; direct evidence of trained innate responses driving diabetic retinopathy remains limited and warrants future investigation.

## Therapeutic targets from trained immunity

**Figure 3:**
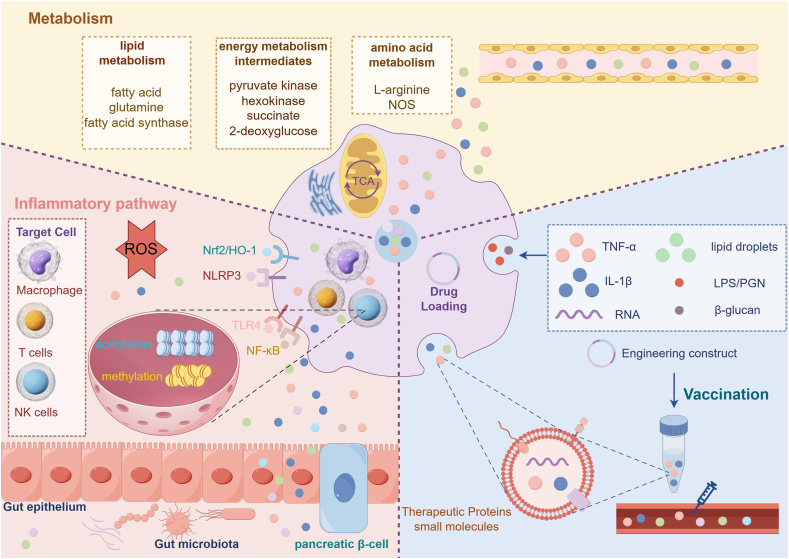
Therapeutic targeting of trained immunity in diabetes. Trained immunity offers novel therapeutic avenues for improving the management of diabetes and its complications. Systemic delivery of therapies targeting trained immune antigens may induce durable memory-like responses in myeloid progenitor cells within the bone marrow. These interventions influence innate immune cells across multiple tissues through epigenetic reprogramming, metabolic remodeling, and immune activation. As a result, heightened immune responses driven by trained immunity can be modulated, thereby reducing inflammation and disease progression. The figure was created using Figdraw.com.

Since TI entails the enduring reprogramming of innate immune cells, leading to improved responses when re-exposed to stimuli, several elements of this mechanism serve as potential therapeutic targets. Gaining insight into and adjusting the pathways that facilitate TI, especially those related to epigenetic and metabolic changes, may offer valuable strategies for both treating and preventing complications associated with diabetes (Fig. 3).

### Focusing on inflammatory pathways

TI is characterized by an intensified inflammatory reaction of the innate immune system upon repeated contact with metabolic stressors or pathogens, which goes beyond classic acute inflammation. This amplified inflammation plays a central role in the pathogenesis of insulin resistance, β-cell dysfunction, along with various diabetes-related vascular complications. Therefore, targeting specific inflammatory pathways activated during TI presents a promising therapeutic strategy. An expanding collection of studies has recognized the *NLRP3* inflammasome as a significant contributor to inflammation associated with diabetes. For example, MCC950, which selectively inhibits *NLRP3*, has been found to diminish islet inflammation and enhance glucose tolerance in a T1D murine model. This outcome correlated with a reduction in *IL-1β* production from islet macrophages displaying TI characteristics.^112^ In a similar vein, Arglabin, another compound that inhibits *NLRP3*, has shown beneficial effects in *ApoE2Ki* mice subjected to a high-fat diet, such as decreased inflammation, maintenance of β-cell health, and slowing T2D progression.^113^ Liu et al further demonstrated in a diabetic atherosclerosis model that using CRISPR/Cas9 to inhibit *NLRP3* in bone marrow-derived macrophages alleviated atherosclerosis *in vivo*. In their study, *NLRP3*-deficient bone marrow-derived macrophages (transplanted into diabetic mice) led to decreased foam cell formation and improved plaque stability, underscoring the contribution of TI to macrovascular complications.^114^ In the context of diabetic kidney disease, Wang et al indicated that isoliquiritigenin, a naturally occurring compound, suppressed *NLRP3* inflammasome activation by blocking the *TLR4/NF-κB/NLRP3* signaling pathway. This action reduces renal inflammation and enhances kidney function in diabetic mice.^115^ Furthermore, triptolide has demonstrated protective effects on podocytes in cases of diabetic kidney disease by *Nrf2*/heme oxygenase 1 (*HO-1*) pathway activation, while concurrently suppressing *NLRP3* inflammasome activation.^116^ Despite the therapeutic potential of direct *NLRP3* inhibitors such as *MCC950*, their clinical development has encountered challenges. As a result, alternative strategies targeting upstream regulators or downstream effectors of NLRP3 are being explored. Inhibitors of *IL-1β*, such as canakinumab, have shown promise in alleviating inflammation and enhancing glycemic control in T2D patients.^117^ Moreover, the sodium-glucose cotransporter 2 (*SGLT2*) inhibitor empagliflozin has been reported to inhibit *IL-17A*-induced proliferation and migration of human aortic smooth muscle cells by modulating the TNF receptor-associated factor 3 (TRAF3) interacting protein 2 (*TRAF3IP2*)*/ROS/NLRP3/caspase-1* pathway, thereby limiting *IL-1β* and *IL-18* secretion.^118^ It should be noted that these persistent epigenetic alterations in retinal cells represent a form of “metabolic memory” in diabetes rather than classical TI of immune cells. Direct evidence implicating trained innate immune cells in diabetic retinopathy is still limited, so the connections discussed here remain speculative.

Elevated levels of ROS trigger multiple inflammatory signaling pathways, including the *NLRP3* inflammasome and *NF-κB*, both of which play a role in intensifying inflammation. Consequently, treatment approaches focused on lowering ROS concentrations or boosting antioxidant defenses could assist in alleviating inflammation linked to TI. N-acetylcysteine, recognized for its strong antioxidant properties, has demonstrated the ability to diminish inflammatory reactions in monocytes that have been exposed to oxidized low-density lipoprotein. This indicates that oxidative stress is crucial in maintaining the TI phenotype.^119^ Additionally, N-acetylcysteine has been reported to alleviate oxidative stress and prevent cell death and skeletal muscle atrophy in T1D via activation of the *Nrf2/HO-1* signaling pathway.^120^ Similarly, tert-butylhydroquinone has demonstrated the ability to reduce podocyte damage in cases of diabetic kidney disease by inhibiting ROS production from *NADPH* oxidase through the *Nrf2/HO-1* pathway.^121^

Apabetalone, a BET protein inhibitor, has been shown to protect the kidneys from diabetes-related injury by preventing pyroptosis through modulation of the *P300/H3K27ac/PLK1* signaling axis.^122^ Zhou et al further demonstrated that the fat mass and obesity-associated protein (*FTO*) reduces TNFAIP3 interacting protein 1 (*TNIP1*) mRNA levels by removing its N6-methyladenosine modification. A decrease in *TNIP1* levels activates *NF-κB* along with various pro-inflammatory pathways, resulting in heightened leakage of retinal blood vessels and the formation of acellular capillaries. Importantly, *TNIP1* expression stabilization through intravitreal administration of an adeno-associated virus considerably reduced endothelial injury. These results emphasize the *FTO–TNIP1–NF-κB* pathway as a promising therapeutic target for addressing diabetic vasculature complications.^123^

### Modulating metabolism

Alongside inflammatory signaling, the reprogramming of metabolism stands out as a crucial aspect of TI, significantly contributing to its initiation and sustenance. This opens up exciting possibilities for therapeutic strategies, especially in tackling the metabolic issues frequently linked to diabetes. For example, promoting oxidative phosphorylation over glycolysis may help attenuate TI-associated inflammation. Metformin, recognized as a primary therapy for T2D, has shown anti-inflammatory effects that could indirectly influence TI by impacting cellular metabolism.^124^ In addition, increasing attention has been directed toward targeting glycolysis in innate immune cells to suppress inflammation. The suppression of essential glycolytic enzymes, such as hexokinase 2 (*HK2*) and pyruvate kinase M2 (*PKM2*), has demonstrated promise in influencing inflammatory responses.^125^^,^^126^ Specifically, *HK2* inhibition has been shown to reduce inflammation in macrophages exposed to high-glucose environments.^127^ An *AMPKβ1*-specific activator reduced inflammation and atherosclerotic progression in diabetic models, illustrating the potential of targeting immune cell metabolism to combat diabetes complications.^128^ *TEPP-46*, an activator of *PKM2* in small-molecule form, has been noted for its ability to restore mitochondrial function and reduce the buildup of harmful glucose byproducts in hyperglycemic environments. The observed effect is linked to an increased glycolytic flux and the up-regulation of peroxisome proliferator-activated receptor-γ coactivator-1α (*PGC-1α*) mRNA in podocytes that have been cultured.^129^ Another potential approach includes utilizing 2-deoxyglucose (*2-DG*), which is a glucose analog that acts as an inhibitor of glycolysis.^130^ Research indicates that *2-DG* enhances insulin sensitivity and diminishes inflammatory responses in *INS-1E* rat insulinoma cells.^131^ However, as *2-DG* broadly suppresses glycolysis, more targeted approaches may be required to minimize off-target effects and improve therapeutic specificity.

Another promising area for treatment involves targeting lipid metabolism in trained innate immune cells. Peroxisome proliferator-activated receptors, especially peroxisome proliferator-activated receptor γ (*PPARγ*), are crucial in lipid metabolism and the inflammatory response.^132^ *PPARγ* agonists, such as thiazolidinediones, have been utilized in T2D management because of their effectiveness in enhancing insulin sensitivity. In addition, thiazolidinediones exhibit anti-inflammatory effects by modulating lipid metabolism in macrophages, which includes promoting fatty acid uptake and storage while reducing the release of inflammatory mediators.^133^, ^134^, ^135^ However, the clinical application of thiazolidinediones is constrained by adverse effects like weight gain and edema, highlighting the need for more selective *PPAR* modulators. Another therapeutic strategy involves targeting fatty acid oxidation (*FAO*). Inhibiting *FAO* in trained immune cells may redirect metabolic processes toward glycolysis, potentially reducing inflammation by limiting the energy available for sustained inflammatory responses.^136^ According to Jones et al, fructose influences glutamine-dependent oxidative metabolism, which in turn supports inflammation triggered by LPS. This finding highlights the connection between metabolic processes and immune system activation.^137^ Additionally, inhibition of lipid synthesis through targeting enzymes such as fatty acid synthase (FASN) has shown promise in reducing inflammatory signaling in macrophages exposed to diabetes-associated stimuli.^138^^,^^139^

The significance of amino acid metabolism in TI in diabetes is becoming a notable field of study. Within the realm of amino acids, l-arginine metabolism is particularly crucial due to its involvement in nitric oxide (NO) production. Nitric oxide synthase (NOS) transforms arginine into NO, which influences both vascular function and inflammatory responses. Under hyperglycemic conditions, TI can disrupt arginine metabolism, leading to excessive NO production that contributes to oxidative stress and vascular dysfunction.^140^ Research involving animals has shown that supplementing with l-arginine can decrease plasma glucose concentrations and enhance glucose tolerance.^141^ Additionally, l-arginine has been found to diminish body fat and improve insulin sensitivity in both obese animal models and people suffering from diabetes and obesity.^142^ Notably, when l-arginine is given alongside metformin, it may be competitively displaced from the NOS activation site, leading to a decrease in NO production.^143^

### Gut microbiome interventions

The gut microbiome is essential for regulating systemic inflammation, insulin resistance, and various metabolic disturbances. Interventions involving probiotics and prebiotics have shown potential in enhancing immune function and mitigating the effects of diabetes.^144^ Prebiotics (inulin and fructo-oligosaccharides) can selectively promote beneficial bacteria growth, including *Bifidobacterium* and *Lactobacillus*.^145^^,^^146^ These bacteria produce short-chain fatty acids, notably butyrate, acetate, and propionate, and are linked to better gut health and immune system regulation.^40^^,^^147^ The addition of dietary inulin led to a decrease in fasting blood glucose and HbA1c levels among T2D individuals.^148^ Furthermore, prebiotics might affect the gut-brain connection by altering neuronal pathways involved in regulating appetite and maintaining glucose balance, in part by promoting the release of incretin hormones.^149^ Numerous clinical studies have emphasized the positive impacts of specific probiotic strains, especially Lactobacillus and Bifidobacterium, on gene expression associated with insulin signaling and inflammation. Research indicates that these probiotics can enhance glycemic regulation and lipid profiles in individuals diagnosed with gestational diabetes.^150^, ^151^, ^152^, ^153^, ^154^ However, the efficacy of probiotics is highly dependent on strain specificity, dosage, and individual host factors, emphasizing the need for personalized approaches. Fecal microbiota transplantation (FMT) has surfaced as a possible treatment. FMT derived from lean donors has demonstrated an improvement in insulin sensitivity among individuals with obesity suffering from metabolic syndrome.^155^ Research conducted by Mocanu et al showed that FMT enhanced glycemic regulation and decreased liver fat levels in individuals suffering from non-alcoholic fatty liver disease alongside T2D.^156^ Nevertheless, findings by Aron-Wisnewsky et al indicated that FMT and probiotic administration from healthy donors did not significantly improve insulin sensitivity or HbA1c levels among T2D individuals.^150^ Consequently, the use of FMT for diabetes is still in the experimental phase, and additional large-scale, randomized controlled studies are necessary to validate its effectiveness and safety.

Diet has a significant impact on the gut microbiome composition. Nutritional choices can either support or impair the growth of beneficial microbial populations, thereby influencing immune and metabolic responses.^157^ An increasing amount of research supports dietary interventions to help regulate blood glucose and prevent diabetes-related complications. For example, a comprehensive review conducted by Garza et al revealed that following the Mediterranean diet, which is marked by a significant consumption of fruits, vegetables, whole grains, and beneficial fats, correlated with better glycemic management and a lower risk of cardiovascular issues in T2D individuals.^158^ Likewise, diets rich in fiber may enhance insulin sensitivity and decrease inflammation among those with prediabetes.^159^ Additionally, foods rich in polyphenols, such as anthocyanins, flavonols, stilbenes, curcuminoids, and phenolic acids, may exert protective effects against diabetes by preserving pancreatic β-cell function, promoting β-cell proliferation, inhibiting apoptosis, and suppressing enzymes involved in carbohydrate metabolism. These compounds also offer antioxidant and anti-inflammatory benefits that support metabolic health and reduce insulin resistance, thereby enhancing pancreatic insulin secretion.^160^, ^161^, ^162^ Given the diversity of gut microbiota and individual metabolic responses to dietary components, personalized nutritional strategies are essential to optimizing therapeutic outcomes in diabetes management.

### Vaccination strategies

The concept of TI presents an intriguing foundation for vaccine-based strategies, particularly in individuals at a higher risk of diabetes and its complications. The BCG vaccine, which was initially for preventing tuberculosis, has shown encouraging results in enhancing TI and aiding glycemic regulation in people with T2D. Several studies have reported that BCG vaccination can lead to sustained reductions in blood glucose and HbA1c levels, along with enhanced insulin sensitivity. These effects are thought to result from epigenetic reprogramming of monocytes, leading to increased *IL-1β* production and the activation of anti-inflammatory pathways.^163^^,^^164^ Research findings from non-obese diabetic (*NOD*) and db/db mice provide additional support for the potential of BCG to lower blood glucose levels and improve glucose tolerance when given in sufficient doses.^165^ Importantly, a randomized clinical trial lasting 8 years, which included patients with chronic T1D, showed that administering two doses of BCG led to nearly normal HbA1c levels after three years, and this enhancement was maintained for five years.^166^ Nevertheless, research conducted by Moghtaderi et al revealed that administering a lower dose of BCG did not produce notable long-term advantages for Iranian individuals with T1D during a span of 48 weeks.^167^ Research in epidemiology has indicated that receiving BCG vaccination during early life might be linked to a lower likelihood of developing both T1D and T2D later on. Nevertheless, this vaccination does not provide the same level of protection against latent autoimmune diabetes in adults.^168^^,^^169^ Furthermore, an independent study revealed that administering a single BCG at nine delayed the onset of T1D by roughly 2.5 years.^170^

The influenza vaccine has also demonstrated potential benefits for patients with diabetes, especially in managing related complications,^171^ beyond its primary role in preventing influenza infection. Vaccination against influenza has been linked to decreased hospitalization rates, especially for conditions like influenza or pneumonia, as well as a reduced overall mortality rate among individuals with diabetes who have received the vaccine, in contrast to those who have not been vaccinated.^172^ These effects are particularly pronounced in elderly patients (aged ≥ 65 years), where seasonal flu vaccination significantly decreases the risk of hospitalization and death.^173^ Current guidelines recommend influenza vaccination for all individuals aged six months and older.^174^ It is important to distinguish between the vaccine's direct protective effects against infection and its broader immunomodulatory impact through TI. Within the framework of T1D, innovative strategies are currently under investigation, such as vaccines based on proinsulin peptides aimed at promoting immune tolerance to islet autoantigens. These vaccines aim to prevent autoimmune-mediated β-cell destruction and potentially delay or prevent disease onset.^175^

## Conclusion

Diabetes and its related complications represent a significant challenge to global health, making it imperative to create new therapeutic strategies. Although conventional methods have mainly concentrated on managing blood sugar levels and alleviating symptoms, increasing research underscores the critical importance of the innate immune system. This system's ability for long-lasting functional reprogramming, referred to as TI, is vital for the development of diabetes and its complications. Therefore, exploring TI within the framework of diabetes signifies a significant change from merely inhibiting inflammation to proactively modifying immune responses to enhance metabolic results. The epigenetic landscape, which governs gene expression patterns, is central to this process. Metabolic stress associated with diabetes induces epigenetic alterations in innate immune cells and promotes a sustained pro-inflammatory state.^176^ Targeted therapies that reverse these epigenetic modifications, such as histone deacetylases or DNA methyltransferase inhibitors, have shown promise in reducing chronic inflammation and restoring immune homeostasis.^57^ An additional significant factor related to TI in diabetes pertains to the metabolic reconfiguration of immune cells. Altered glucose and lipid metabolism, driven by hyperglycemia and insulin resistance, enhances inflammatory cytokine production and impairs immune regulation.^177^ Interventions aimed at modifying metabolic pathways present opportunities to diminish inflammation. For example, *2-DG* application to inhibit glycolysis has demonstrated a reduction in cytokine levels and an enhancement in insulin sensitivity.^178^ While conventional vaccines are designed to stimulate adaptive immunity, recent studies suggest that modified BCG vaccines and other innate immune activators may induce TI with beneficial effects on metabolic function. These vaccines have been associated with enhanced glucose uptake and reduced systemic inflammation, offering a novel immunometabolic approach to diabetes management.^179^ Nonetheless, additional studies are required to enhance these approaches and evaluate their long-term safety and effectiveness.

In summary, TI provides a promising framework for rethinking the mechanisms underlying diabetes and its complications. By clarifying the epigenetic and metabolic foundations of innate immune memory and pinpointing specific therapeutic possibilities, it could be feasible to reestablish immune–metabolic equilibrium and enhance clinical results. Future efforts could prioritize translating these findings into clinical trials and developing personalized therapeutic strategies that address individual variations in immune programming and disease phenotype. In addition, integrating conventional treatments with TI-targeted interventions could ultimately unlock the full potential of the immune system in fighting against diabetes and its long-term consequences.

## CRediT authorship contribution statement

**Qiming Gong:** Writing – original draft, Visualization, Project administration, Conceptualization. **Yuqing Huang:** Writing – original draft. **Fahui Liu:** Writing – original draft. **Tingting Zhou:** Writing – original draft. **Wei Huang:** Writing – review & editing, Funding acquisition. **Yong Xu:** Writing – review & editing, Funding acquisition.

## Funding

This work was supported by grants from the 10.13039/501100001809Natural Science Foundation of China (No. 82170834, U22A20286, 82470854), Sichuan Science and Technology Program (China) (No. 2024YFFK0081), Sichuan Province Cadre Health Research Project (China) (No. ZH2022-1501), Health Commission of Sichuan Province Medical Science and Technology Program (China) (No. 24CXTD02), Clinical Medicine Special Project of Southwest Medical University (Sichuan, China) (No.2024LCYXZX02, 2024LCYXZX12), and Graduate Education and Teaching Program of Southwest Medical University (Sichuan, China) (No. YJG202291, ZYTS-29).

## Conflict of interests

No conflict of interests, financial or otherwise, is declared by the authors.
